# Phase Equilibrium Calculation Method and Phase Equilibrium Curve Characterization of Natural Gas Hydrates Under the Action of Polymer Additives in Cement Slurry Filtrate: Based on Molecular Dynamics Simulation

**DOI:** 10.3390/ma19050858

**Published:** 2026-02-25

**Authors:** Huajie Liu, Wenxiang Lin, Sergey E. Chernyshov, Theis I. Solling, Xinyue Zhao, Zhiwei Tao

**Affiliations:** 1 State Key Laboratory of Deep Oil and Gas, China University of Petroleum (East China), Qingdao 266580, China; z23020064@s.upc.edu.cn (W.L.);; 2 School of Petroleum Engineering, China University of Petroleum (East China), Qingdao 266580, China; 3 Shandong Key Laboratory of Offshore Oil & Gas and Hydrates Development, Qingdao 266580, China; 4 Oil and Gas Technologies Department, National Research Polytechnic University, 614990 Perm, Russia; 5 Petroleum Engineering Department, King Fahd University of Petroleum and Minerals-KFUPM, Dhahran 31261, Saudi Arabia

**Keywords:** natural gas hydrate, well cement slurry, polymer additives, phase equilibrium calculation method, phase equilibrium curve characterization

## Abstract

Polymer additives in well cement slurry filtrate would affect the stability of natural gas hydrate (NGH), which could lead to formation collapse and cause marine disasters. It is necessary to clarify the critical conditions for the stability of NGH, i.e., NGH phase equilibrium. LAMMPS software and the TIP4P model were used to develop a method for calculating NGH phase equilibrium. Based on the single functional groups and combined functional groups of polymer additives, the potential energy, angular order parameter (AOP) of water molecules, and NGH phase equilibrium temperatures under different pressures were calculated, and a phase equilibrium curve was characterized. Results show that amide groups promote NGH decomposition more strongly than carboxyl and sulfonate groups, with a 1.5% dodecylamide system causing NGH phase equilibrium temperature to decrease by 1.68–2.77 K. AM/AA promotes NGH decomposition more strongly than AA/AMPSNa, AM/AMPSNa, and AA/AMPSNa/AM, with a 1.5% AM/AA system causing NGH phase equilibrium temperature to decrease by 2.33–3.56 K. To ensure the safety of well cementing and marine environments, the contents of amide groups and carboxyl groups should be reduced when developing polymer additives for cement slurry used in NGH formation cementing.

## 1. Introduction

Deep-water natural gas hydrate is a kind of ice-cage-like crystal formed by CH_4_-based hydrocarbon gas and H_2_O under high pressure and low temperature conditions. As a clean and efficient resource with huge reserves, NGH is widely distributed in the permafrost zone of high-latitude continental areas and deep-sea sedimentary rocks, which is a future alternative energy source with great potential [1,2,3]. However, the drilling and development of NGH is still in its infancy, facing problems such as high safety risk, high extraction cost, and imperfect technology [4,5], and the cementing of NGH is very important as one of the key aspects of NGH test mining operation [6,7]. The temperature and pressure fluctuations induced by cementing operations in NGH-containing formations can potentially induce NGH decomposition, leading to significant gas generation and issues like annular air scrambling. These events can compromise the stability and load-bearing capacity of the NGH, impacting the wellhead stability during deep-water drilling, thereby posing a serious threat to the safety of cementing operations [8].

Research on wellbore stability during cementing in NGH formations primarily concentrates on the advancement of low hydration hot water mud systems. Two main strategies are commonly employed: the first involves diminishing the liberation of hydration heat during the waiting period of the cement slurry by incorporating low-heat materials [9], while the second entails absorbing a portion of the cement hydration heat through the addition of phase change energy storage materials [10,11]. In addition to changes in the temperature of the NGH caused by cement hydration, polymer additives with a specific structure are also one of the factors affecting the stability of NGH [12,13], and different structures of polymer additives have different effects on NGH, and it has been found that according to the different effects of the role of the polymer additives can be divided into four kinds of inhibitors of NGH generation, NGH generation promoters, NGH decomposition promoters, and NGH decomposition inhibitors [14,15,16]. Therefore, the effect of polymer additives of different structures on the stability of NGH must be studied when the configuration of cement slurry systems is carried out.

The filtrate from cement slurry contains a significant quantity of polymer additives and ions. As the cement slurry waits to set, filtration loss occurs, allowing free polymer additives and ions to permeate the formation. Huo [17] et al. studied the effects of inorganic ions and single functional groups on the stability of NGH. Results showed that inorganic ions had little effect on the stability of NGH; NGH decomposition was the fastest under the action of amide groups in a single functional group; and the diffusion coefficient of water molecules under the action of dodecyl amide increased by 23.82–40.86% compared with the pure water–NGH system. Lin [18] et al. studied the effect of polymers in cement slurry filtrate on the stability of NGH. Those results showed that the AM/AA–hydrate system had the fastest NGH decomposition rate at the same solute concentration and that the diffusion coefficient of water molecules was increased by 39.01% to 123.96% compared with that of the pure water–NGH system. Polymer additives in cement slurry filtrate would inevitably influence NGH stabilization.

Therefore, it is necessary to clarify the critical conditions of NGH stability under the influence of polymer additives, but there lacks a phase equilibrium calculation method and phase equilibrium curve characterization for NGH. In this study, LAMMPS software (20210401) and the TIP4P model were used to develop a method for calculating NGH phase equilibrium. Based on the single functional groups and combined functional groups in polymer additives, the potential energy, angular order parameter of water molecules, and NGH phase equilibrium temperatures under different pressures were calculated. From the calculation results, the critical conditions for NGH stability were determined, and the phase equilibrium curve was characterized. The research provides a design basis for polymer additives used in cement slurry for NGH formation cementing.

## 2. Modeling Methodology and NGH Phase Equilibrium Calculation Method Design

### 2.1. Molecular Force Field Parameters for Modeling NGH Multiphase Systems

The TIP4P model has a high calculation accuracy in terms of phase changes. Compared with other software, LAMMPS software is more flexible in setting potential energy models such as TIP4P models, and has high computational efficiency, which is suitable for simulation calculations on large spatiotemporal scales. Therefore, the simulations were studied by LAMMPS software.

**Figure 1:**
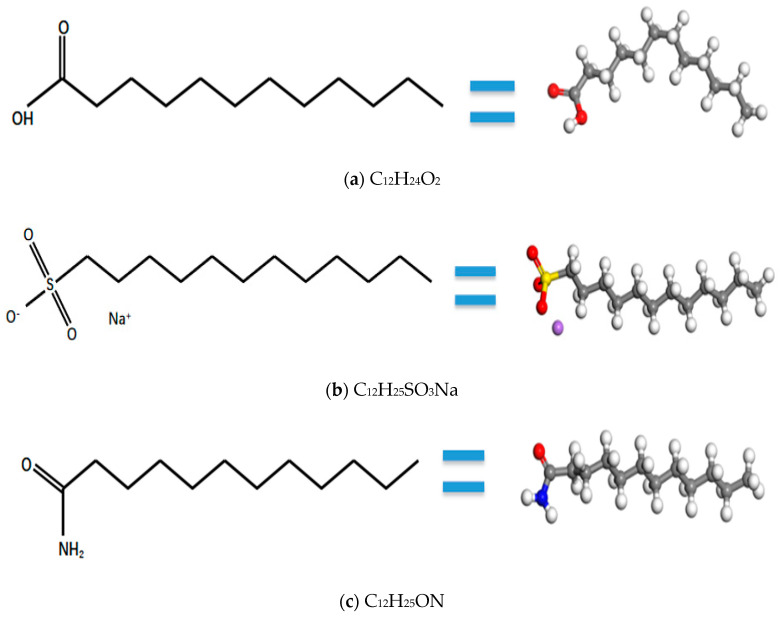
Molecular structure of a single functional group. The red ball is the oxygen atom, the white ball is the hydrogen atom, the gray ball is the carbon atom, the blue ball is the nitrogen atom, the yellow ball is the sulfur atom, and the purple ball is the sodium ion.

Previous studies [17,18,19] have identified molecular models of single and combined functional groups and investigated their effects on NGH stability. The single functional groups are C_12_H_24_O_2_, C_12_H_25_SO_3_Na, and C_12_H_25_ON, and the molecular model is shown in Figure 1.

The selection of combined functional groups was based on practical production considerations, choosing representative synthetic monomers for cementing organic additives: 2-acrylamido-2-methylpropanesulfonic acid (AMPS), acrylic acid (AA), and acrylamide (AM). These three components can form four alternating copolymer structures: AM/AA, AA/AMPS, AM/AMPS, and AA/AMPS/AM. Infrared spectroscopy analysis [17] revealed that the primary functional groups in the cementing additive were carboxyl, amide, and sulfonic acid groups. Therefore, sodium 2-acrylamido-2-methylpropanesulfonate (AMPSNa) was selected as the monomer, with AM/AA, AA/AMPSNa, AM/AMPSNa, and AA/AMPSNa/AM being the research subjects.

This paper adopts the molecular model established in previous studies [18], which has structural characteristics of the filtrate components. The core parameters of the OPLS-AA force field reflect the intrinsic properties of molecules and are not sensitive to moderately high pressures. The effect of increased molecular density under high pressure is accurately handled through periodic boundary conditions and the PPPM algorithm. Therefore, the force field parameters for water molecules are based on the original TIP4P model (Table 1), with the charge of the M site equivalently assigned to the oxygen atom (O), methane molecules to the OPLS-AA model (Table 2), and solute molecules to the OPLS-AA model (Table 3). The charge distributions of molecules modeled with the OPLS-AA force field model were adjusted following the 1.14*CM1A charge model [20,21].

### 2.2. Modeling of NGH Multiphase Systems

Based on previous research findings [17,18], there are significant differences in the diffusion properties of various organic molecule solution-hydrate systems within the range of 0.5–1.5%, while the differences are smaller within the range of 1.5–2.5%. Therefore, the range of 0.5–1.5% is selected for simulation calculations. A total of 22 models were built to investigate the diffusion properties of organic molecule solution–NGH systems within the 0.5–1.5% range. These models included variations such as pure water–NGH layer, C_12_H_24_O_2_ solution–NGH layer, C_12_H_25_SO_3_Na solution–NGH layer, C_12_H_25_ON solution–NGH layer, AM/AA solution–NGH layer, AA/AMPSNa solution–NGH layer, AM/AMPSNa solution–NGH layer, AA/AMPSNa/AM solution–NGH layer, and AMPSNa/AM solution–NGH layer. The outcomes of the model conversion were imported into the visualization software OVITO 3.5.0, revealing the initial configuration of the model featuring a solute–solvent substance ratio of 0.5%.

### 2.3. NGH Phase Equilibrium Simulation Calculation Method

With reference to the experimental determination of NGH phase equilibrium using the direct observation method, which was used to design the phase equilibrium simulation of the solution–NGH system, the simulation process involves maintaining constant pressure while gradually increasing the temperature. By observing the pressure at which NGH formation is detected and continuing to raise the temperature until complete decomposition of NGH occurs, the temperature at which this decomposition happens represents the equilibrium temperature of the NGH phase under that pressure. According to the research literature [22,23], the stabilization temperatures for NGH drilling sites SH2, SH3, and SH7 in the Shenhu Sea area of the northern part of the South China Sea were determined to be 277.99 K, 278.68 K, and 279.59 K, respectively (corresponding to NGH phase equilibrium pressures <5.02 MPa). Since the seafloor pressure exceeds 5.02 MPa, NGH stabilization is achievable. Because the pressure in the actual marine environment is changing, it is necessary to simulate a number of different pressure values to comprehensively understand the phase equilibrium characteristics of NGH under different pressure conditions, and explore the influence of pressure on the phase equilibrium temperature and other properties of NGH, so as to accurately simulate and study the actual existence state and phase equilibrium behavior of NGH in the Shenhu area and other similar environments in the northern part of the South China Sea. Therefore, considering the NGH data from drilling areas and phase equilibrium data in the South China Sea, system pressures of 5.02 MPa, 7.59 MPa, 8.55 MPa, 9.17 MPa, and 10.57 MPa have been selected for the simulation in this study.

The basic elements of the phase equilibrium simulation calculation method are as follows [24]:

(1) Periodic boundary conditions are adopted in the X, Y, and Z directions to reduce the edge effect and simplify the physical problem with spatial periodicity to a small basic unit, so as to simulate the infinite real environment. The selection of the truncation radius is of great significance to the accuracy of the calculation, and for the system with periodic boundary orthogonal boxes, the truncation radius is usually set to half of the length of the shortest side of the box, which can not only ensure a certain calculation accuracy, but also avoid the repeated calculation caused by the interaction between the calculated atoms and the mirror image, so the truncation radius is 1 nm, and the Particle–Particle Particle–Mesh (PPPM) algorithm is utilized for handling long-range interactions, with periodic storage managed through the restart command. The size of the time step is generally selected as one-tenth of the minimum vibrational period of particle motion, and among organic molecules, the expansion and contraction vibration period of carbon–hydrogen bonds is the shortest. Therefore, the initial model configuration undergoes energy minimization and a relaxation period of 1 ns under the NVT ensemble. Following the relaxation phase, the simulation transitions to the NPT ensemble for incremental temperature elevation, with a time step of 1 fs. Each temperature increment involves a simulation duration of 1 ns to achieve equilibrium at the given temperature, continuing until complete decomposition of NGH is achieved. The thermo command is used to output system potential energy, system temperature, as well as the trajectory and equil files.
(2) The output trajectory is examined and the Angular Order Parameter (AOP) of water molecules is determine at the point where the cage structure dissipates entirely and the system’s potential energy stabilizes. According to [25], the AOP is 0.1 for NGH crystals and 0.8 for liquid water. When the AOP exceeds the 0.8 threshold, it signifies the complete decomposition of NGH.
(3) The thermo command outputs the total system temperature every 0.005 ns for calculating the arithmetic average temperature change curve. This data is then used to analyze the relationship between temperature variations over time by performing a fourth-degree polynomial fitting to derive the fitted temperature curve. The moment of complete decomposition of NGH corresponds to the temperature value obtained from this fitting, representing the simulated phase equilibrium temperature.

### 2.4. Calculation Method Example and Reasonableness Verification

**Figure 2:**
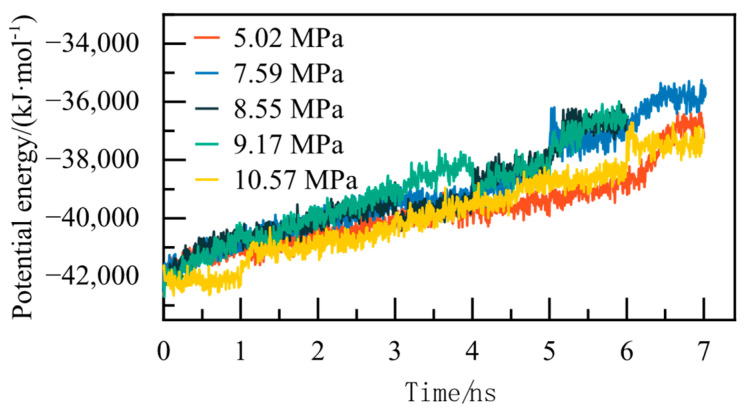
Potential energy changes at different pressures.

**Figure 3:**
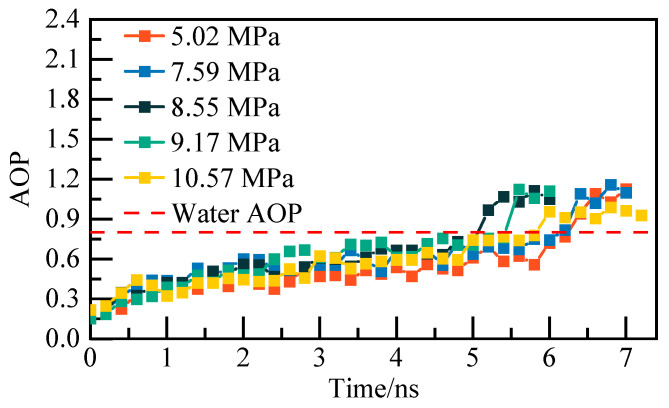
AOP changes of water molecules at different pressures.

The reliability of the phase equilibrium temperature calculation method is demonstrated using the pure water–NGH system model as a case study. Initially, the potential energy and Angular Order Parameter (AOP) of the system are computed under varying pressures to identify the specific moment of complete NGH decomposition. The potential energy calculation results are illustrated in Figure 2, while the AOP calculation findings are displayed in Figure 3. The potential energy curves indicate that as NGH decomposes, the system’s potential energy tends to rise, with a sudden transition to a stable state as NGH nears complete decomposition. Moreover, the AOP curves reveal that the AOP of water molecules fluctuates above 0.8, indicating a full transformation into liquid water. Once the AOP reaches 0.8, the water molecules have entirely converted into a liquid state. By integrating the potential energy curve and the AOP curve, it is evident that NGH reaches complete decomposition when the system’s potential energy abruptly stabilizes and the AOP reaches 0.8.

**Figure 4:**
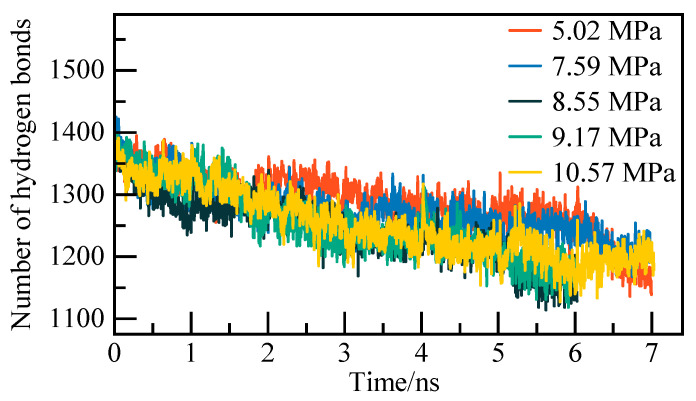
Diagram of the number of hydrogen bonds.

The evolution of hydrogen bonding and snapshots of the system simulation process are examined to determine the precise moment of complete NGH decomposition. The system’s hydrogen bonding count was computed, and a snapshot of the system simulation process was observed for the pure water–NGH system at 5.02 MPa, serving as an illustrative example. The outcomes of the hydrogen bond number calculation are depicted in Figure 4, the decomposition of NGH leads to the rupture of hydrogen bonds between water molecules, resulting in a decrease in the system’s total hydrogen bonds during the phase transition. Subsequently, the system attains a new equilibrium state. Throughout NGH phase transition, as decomposition intensifies, methane and water molecules within the crystal gradually separate. The water molecules transition from an ordered arrangement to disordered liquid form, while the distance between methane molecules expands, diffusing towards the liquid water film on both sides. By 6.4 nanoseconds, the cage structure of NGH is entirely dismantled, aligning with the phase transition indicated by the potential energy curve and AOP curve.

After identifying the instant of complete NGH decomposition, to investigate the correlation between temperature and time and forecast NGH phase equilibrium temperature, a fourth-order polynomial was utilized to fit the average temperature curves at various pressures. The fitting equation is presented as Equation (1):

(1) $$T=A+B_{1}t^{1}+B_{2}t^{2}+B_{3}t^{3}+B_{4}t^{4}$$

In (1), T is the fitted temperature, K; t is the simulation time, ns; A\$B_{1}$\$B_{2}$\$B_{3}$\$B_{4}$ are constants.

**Figure 5:**
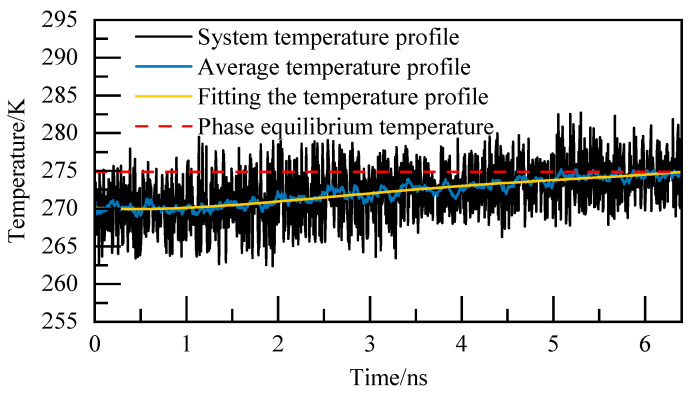
Temperature curves at 5.02 MPa.

For instance, at 5.02 MPa, as depicted in Figure 5, the complete decomposition of NGH occurs precisely at 6.4 nanoseconds, marking the phase equilibrium temperature at 274.86 K. This temperature of 274.86 K for NGH is clearly indicated in Figure 5.

**Figure 6:**
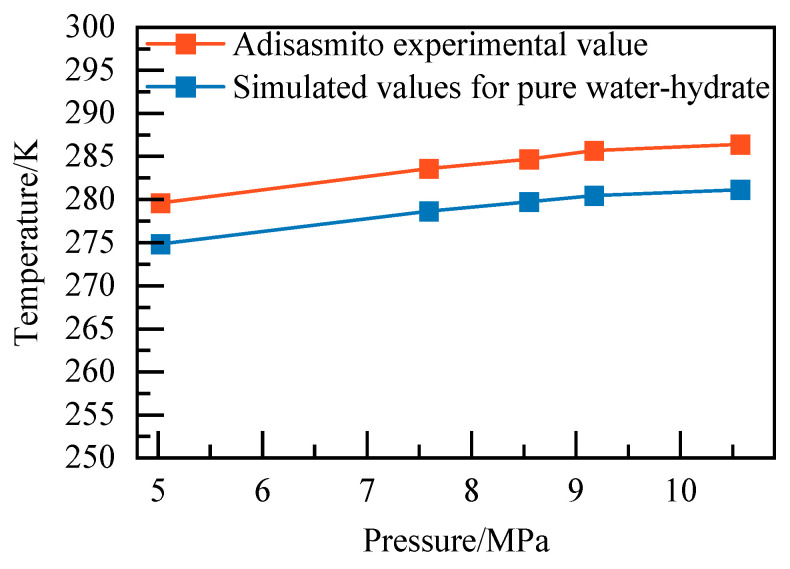
Diagram of NGH phase equilibrium.

The results of the temperature calculations for the pure water–NGH system under different pressures are shown in Table 4. The calculated NGH phase equilibrium conditions are compared with NGH phase equilibrium conditions in the literature [25], as shown in Figure 6. Due to the difference between the ideal system of the molecular simulation and the actual system, the relative error of the simulated value of NGH phase equilibrium temperature is 1.70–1.84%, and the simulated value of the phase equilibrium temperature increases as the system pressure increases. When value increases, the absolute error between the simulated and experimental values has been stable at 5 ± 0.26 K. This error is a systematic error, arising from the inherent simplifications of the model and force field, and is independent of pressure while remaining within an acceptable range. Therefore, the NGH phase equilibrium simulation calculation method used is reliable.

## 3. NGH Phase Equilibrium Conditions and NGH Phase Equilibrium Curve Characterization

### 3.1. NGH Phase Equilibrium Conditions and NGH Phase Equilibrium Curve Characterization Under the Action of a Single Functional Group

#### 3.1.1. C_12_H_24_O_2_

**Figure 7:**
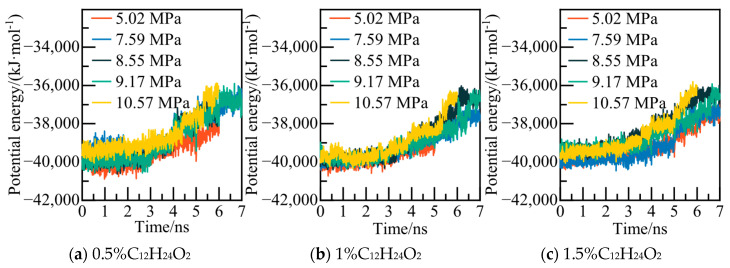
Potential energy changes at different pressures.

**Figure 8:**
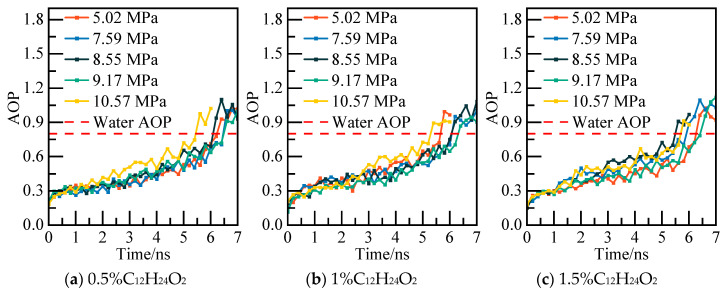
AOP changes of water molecules at different pressures.

The calculated potential energy of the C_12_H_24_O_2_ system and the AOP values of water molecules under various pressures are displayed in Figure 7 and Figure 8. Taking the 0.5% C_12_H_24_O_2_ system model under 5.02 MPa as an example, at 6.4 ns, the potential energy of the system exhibits slight fluctuations in an equilibrium state. The calculated AOP value exceeds 0.8, indicating that all water molecules in the system have converted to liquid water to achieve a new equilibrium. At this juncture, it is deemed that NGH has undergone complete decomposition.

**Figure 9:**
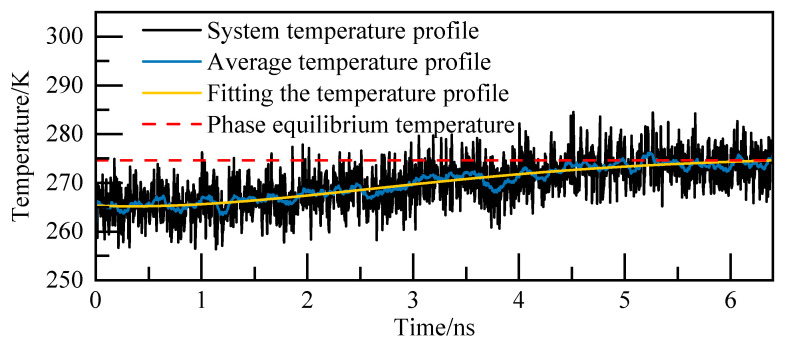
Temperature curves at 5.02 MPa.

For the 0.5% C_12_H_24_O_2_ system at 5.02 MPa, the phase equilibrium temperature of NGH was calculated. The temperature profile, as depicted in Figure 9, was analyzed in conjunction with the potential energy profile and AOP profile. It was determined that NGH underwent complete decomposition at 6.4 ns, with the phase equilibrium temperature recorded at 274.6 K. The temperature of NGH was calculated through this analysis.

**Figure 10:**
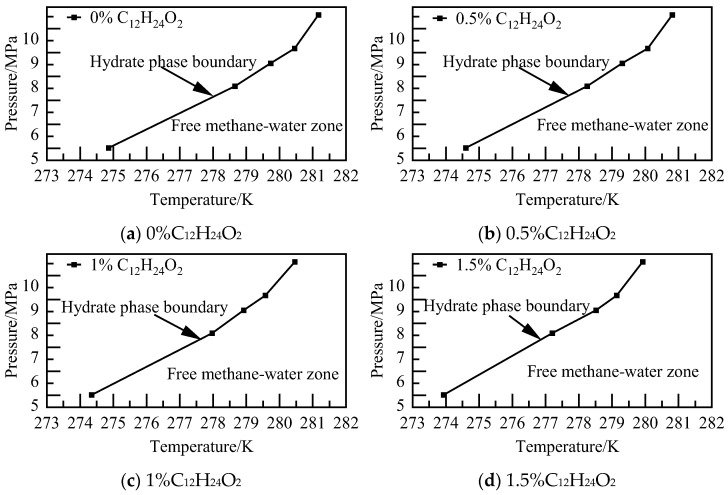
Diagram of NGH phase equilibrium.

The findings from the phase equilibrium calculations for C_12_H_24_O_2_ systems at varying concentrations and pressures are illustrated in Figure 10. It was observed that NGH phase equilibrium shifted towards lower temperatures with increasing solution concentrations. Specifically, NGH phase equilibrium temperature of the 1.5% C_12_H_24_O_2_ system decreased by 0.93–1.46 K in comparison to that of the pure water–NGH system.

#### 3.1.2. C_12_H_25_SO_3_Na

**Figure 11:**
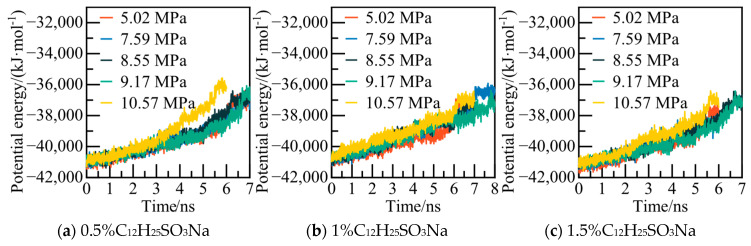
Potential energy changes at different pressures.

**Figure 12:**
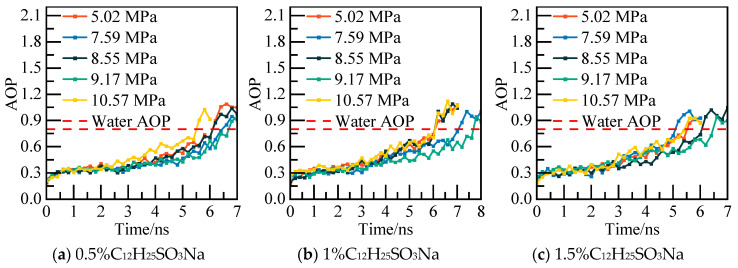
AOP changes of water molecules at different pressures.

The potential energy and AOP values of water molecules in the C_12_H_25_SO_3_Na system under various pressures were calculated, as depicted in Figure 11 and Figure 12. Specifically, for the 0.5% C_12_H_25_SO_3_Na system at 5.02 MPa, the potential energy of the system reached a new equilibrium state at 6.2 ns. Simultaneously, the AOP value reached the standard of liquid water, indicating complete decomposition of NGH.

**Figure 13:**
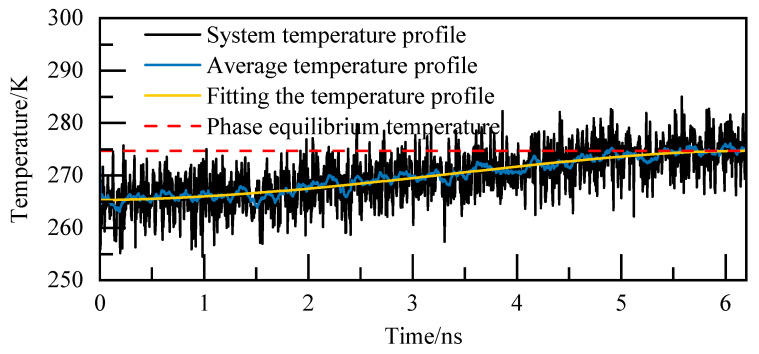
Temperature curves at 5.02 MPa.

The temperature profile for the 0.5% C_12_H_25_SO_3_Na system at 5.02 MPa was calculated, and the results are presented in Figure 13. NGH phase equilibrium temperature was determined to be 274.73 K based on the data from the temperature profile. This temperature represents the point at which NGH phase equilibrium is achieved in the system.

**Figure 14:**
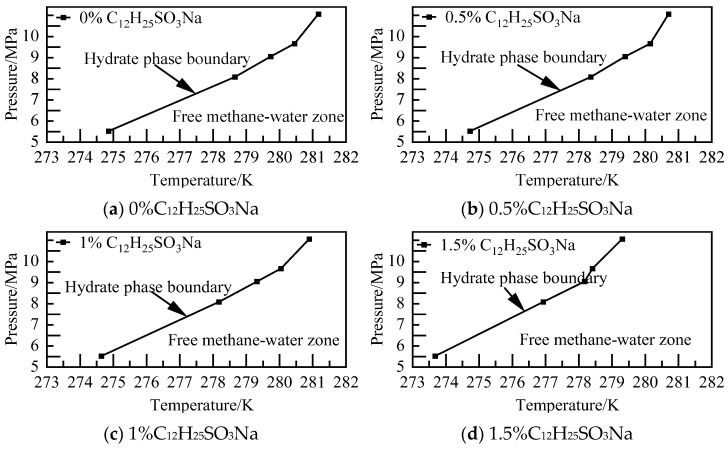
Diagram of NGH phase equilibrium.

The temperature calculations for various concentrations of C_12_H_25_SO_3_Na systems at different pressures are depicted in Figure 14. It was observed that at lower concentrations, the solution has a minor impact on the phase equilibrium of NGH. However, as the concentration increases, the region where NGH can be stabilized decreases. Additionally, the temperature of the phase equilibrium of NGH in the 1.5% C_12_H_25_SO_3_Na system decreases by 1.18–2.04 K compared to that of the pure water–NGH system.

#### 3.1.3. C_12_H_25_ON

**Figure 15:**
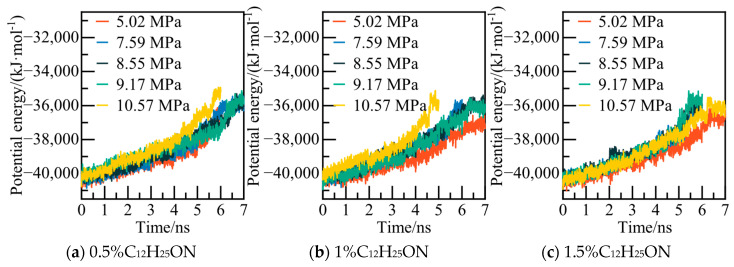
Potential energy changes at different pressures.

**Figure 16:**
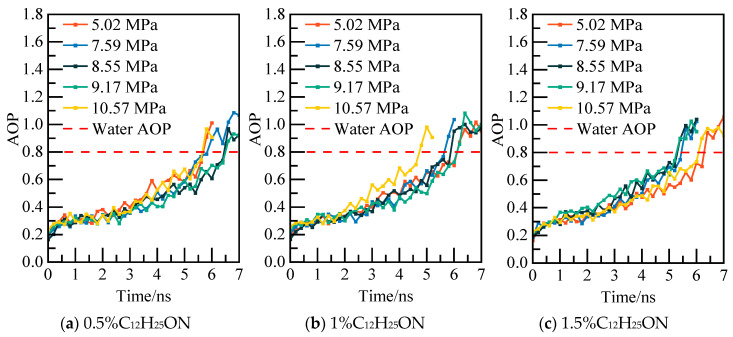
AOP changes of water molecules at different pressures.

The potential energy and water molecule AOP values of the C_12_H_25_ON system at various pressure values are illustrated in Figure 15 and Figure 16. For instance, in the case of the 0.5% C_12_H_25_ON system at 5.02 MPa, the potential energy is re-equilibrated at 5.8 ns. The calculated water molecule AOP value fluctuates at a level higher than 0.8, indicating that NGH is completely decomposed at that point in time. This information provides insights into the behavior and stability of NGH system under different conditions.

**Figure 17:**
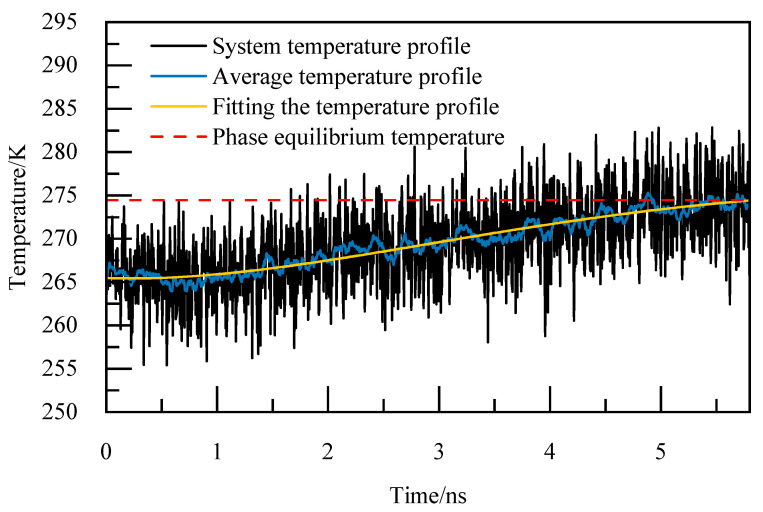
Temperature curves at 5.02 MPa.

In the case of the 0.5% C_12_H_25_ON system at 5.02 MPa, the calculated temperature profile is depicted in Figure 17. By analyzing this temperature profile in conjunction with the potential energy curve and AOP curve, it was determined that NGH undergoes complete decomposition at 5.8 ns. At this moment, the temperature of NGH phase equilibrium was read to be 274.44 K. This information provides valuable insights into the behavior of the NGH system and its stability under specific conditions.

**Figure 18:**
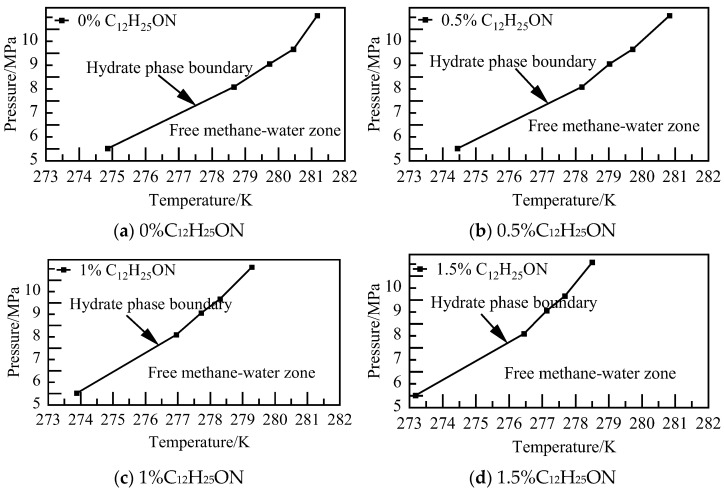
Diagram of NGH phase equilibrium.

The results of phase equilibrium calculations for different concentrations of the C_12_H_25_ON system at various pressures are depicted in Figure 18. As the concentration of C_12_H_25_ON increases, NGH phase equilibrium shifts towards lower temperatures, resulting in a decrease in NGH phase equilibrium temperature of the 1.5% C_12_H_25_ON system by 1.68–2.77 K compared to that of the pure water–NGH system.

For carboxyl groups, sulfonic acid groups, and amide groups under the same conditions, the amide groups promote the decomposition of NGH more strongly than the carboxyl and sulfonic acid groups.

### 3.2. NGH Phase Equilibrium Conditions and NGH Phase Equilibrium Curve Characterization Under the Synergistic Action of Functional Groups

#### 3.2.1. AM/AA

**Figure 19:**
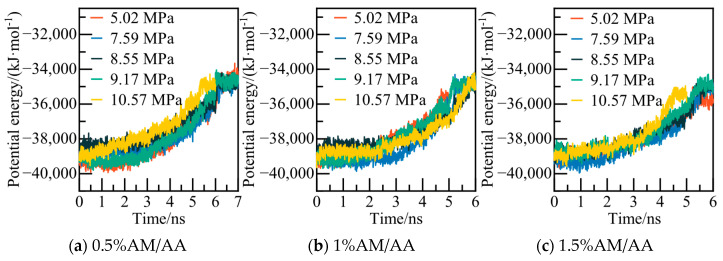
Potential energy changes at different pressures.

**Figure 20:**
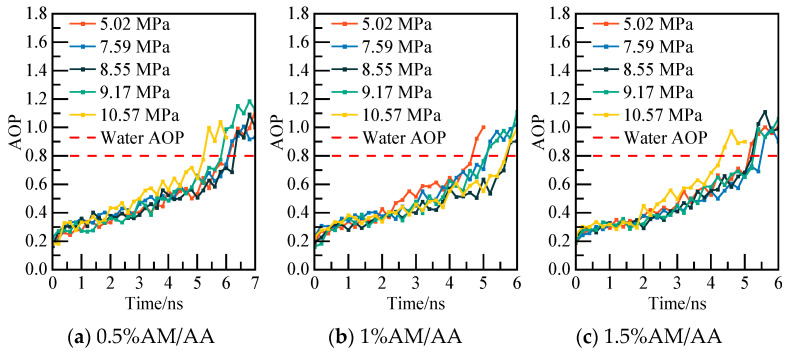
AOP changes of water molecules at different pressures.

The calculated potential energy of the AM/AA system and the AOP value of water molecules are presented in Figure 19 and Figure 20, respectively. Using the 0.5% AM/AA system model at 5.02 MPa as a case study, the system’s potential energy stabilizes at 6.2 ns, with the AOP value exceeding 0.8 at this stage, indicating complete decomposition of NGH.

**Figure 21:**
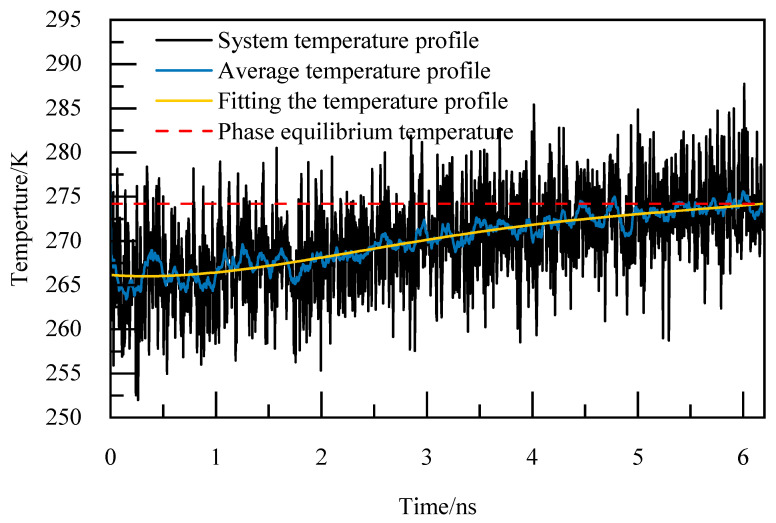
Temperature curves at 5.02 MPa.

The calculated temperature profile for the 0.5% AM/AA system at 5.02 MPa is illustrated in Figure 21. This profile, when analyzed alongside the potential energy profile and AOP profile, indicates the complete decomposition of NGH at 6.2 ns. The phase equilibrium temperature of NGH is determined to be 274.21 K at this pressure.

**Figure 22:**
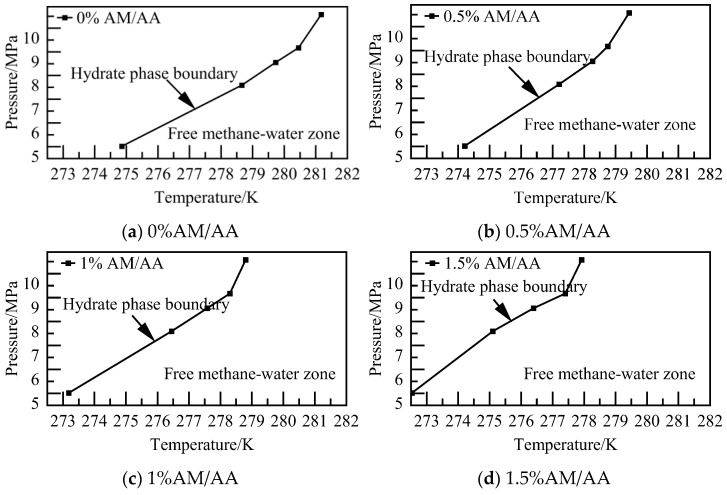
Diagram of NGH phase equilibrium.

The phase equilibrium calculations for the AM/AA system with varying concentrations at different pressures are characterized in Figure 22. As the concentration increases, the stable region where deep-water hydrate crystals can exist diminishes. The phase equilibrium of NGH shifts towards lower temperatures, with NGH phase equilibrium temperature of the 1.5% AM/AA system decreasing by 2.33–3.56 K compared to that of the pure water–NGH system.

#### 3.2.2. AA/AMPSNa

**Figure 23:**
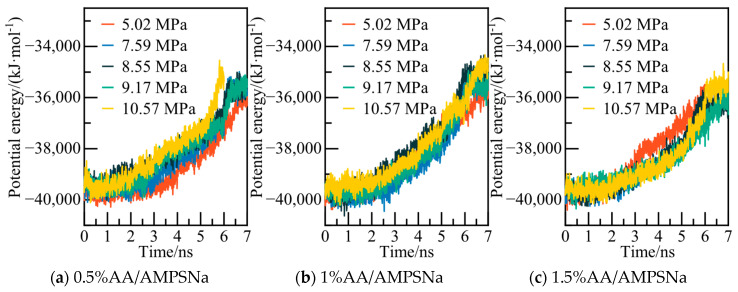
Potential energy changes at different pressures.

**Figure 24:**
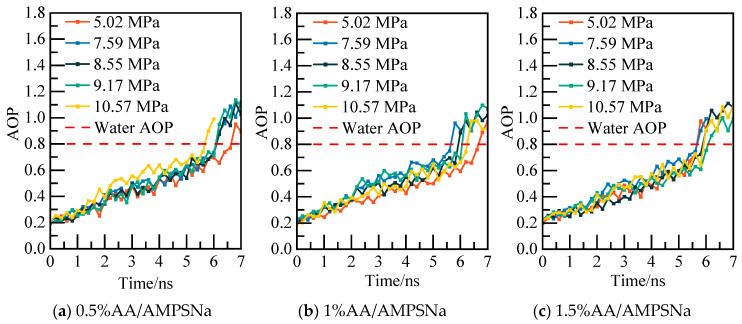
AOP changes of water molecules at different pressures.

The potential energy and water molecules AOP value for the AA/AMPSNa system are depicted in Figure 23 and Figure 24, respectively. Under a pressure of 5.02 MPa in the 0.5% AA/AMPSNa system model, at 6.8 ns, the system undergoes complete conversion of liquid water, leading to the potential energy curve reaching a steady state. This indicates that the system has reached a new equilibrium, signifying that NGH has been completely decomposed at this point.

**Figure 25:**
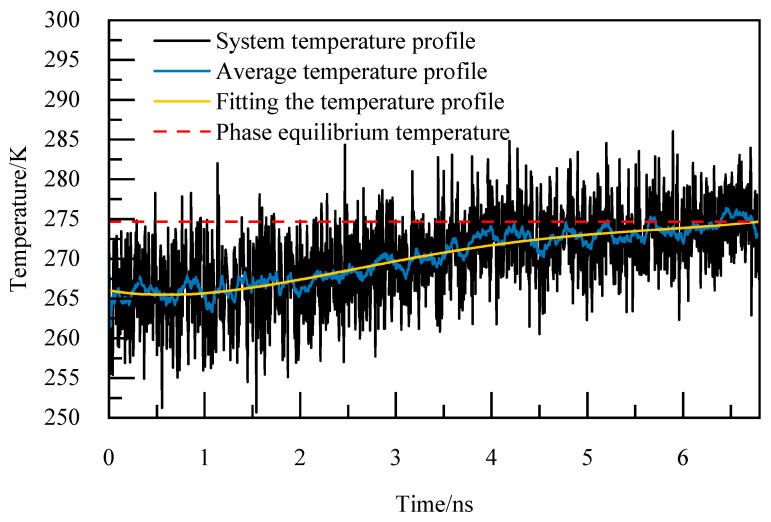
Temperature curves at 5.02 MPa.

In the case of the 0.5% AA/AMPSNa system model at 5.02 MPa, the temperature profile is displayed in Figure 25. By integrating this information with the potential energy curve and AOP curve, the complete decomposition of NGH is determined to occur at 6.8 ns, with NGH phase equilibrium temperature reading at 274.68 K.

**Figure 26:**
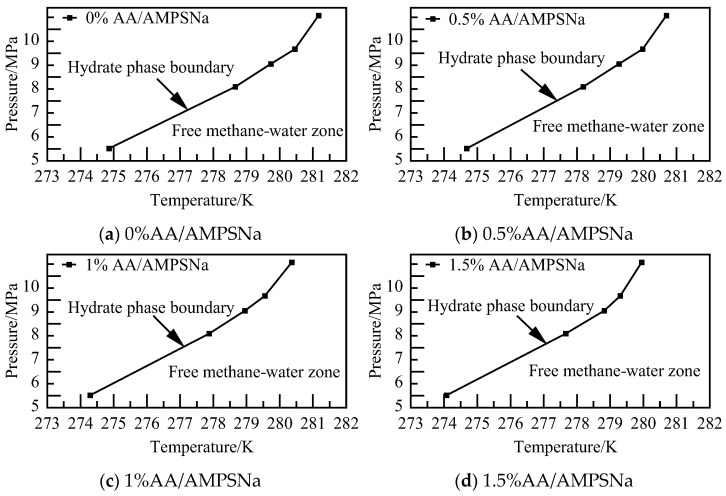
Diagram of NGH phase equilibrium.

The phase equilibrium calculations for the AA/AMPSNa system at various concentrations and pressures are illustrated in Figure 26. It is observed that NGH phase equilibrium temperature decreases with an increase in solution concentration. Specifically, NGH phase equilibrium temperature for the 1.5% AA/AMPSNa system is lower by 0.79–1.22 K compared to that of the pure water–NGH system.

#### 3.2.3. AM/AMPSNa

**Figure 27:**
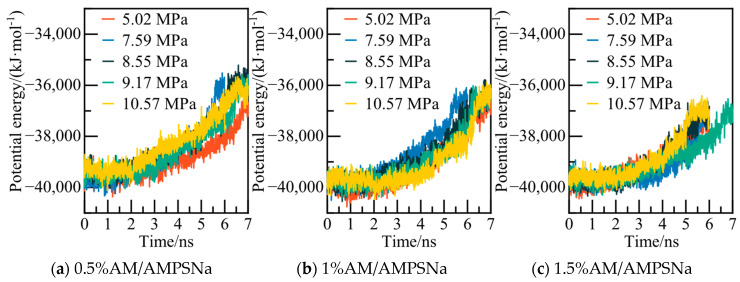
Potential energy changes at different pressures.

**Figure 28:**
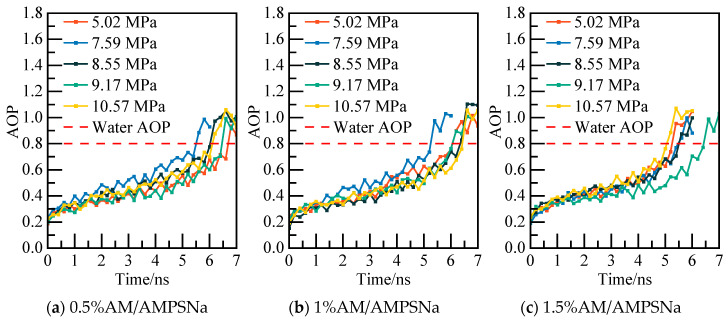
AOP changes of water molecules at different pressures.

The potential energy and water molecule AOP values for the AM/AMPSNa system are illustrated in Figure 27 and Figure 28. Taking the 0.5% AM/AMPSNa system model at 5.02 MPa as an example, as NGH decomposition progresses, the system’s potential energy increases. At 6.8 ns, the potential energy reaches equilibrium, and the corresponding AOP values indicate that all water molecules in the system have transitioned into liquid water molecules. This signifies the complete decomposition of NGH.

**Figure 29:**
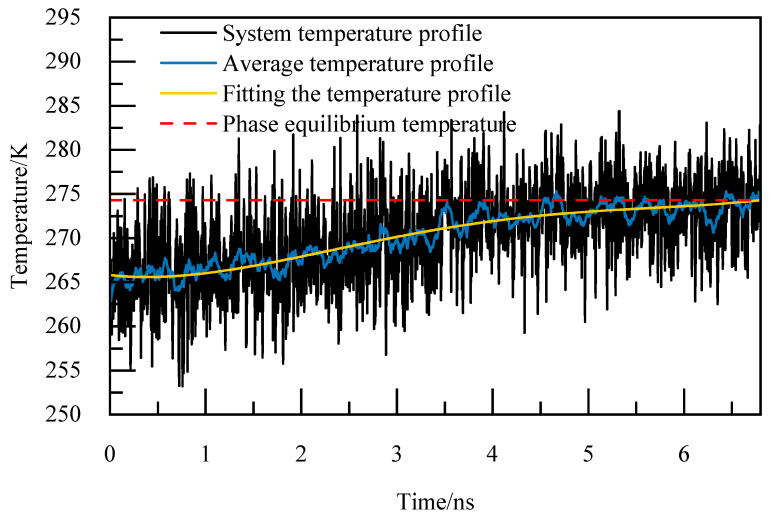
Temperature curves at 5.02 MPa.

An example of the calculated temperature profile for the modeled 0.5% AM/AMPSNa system at 5.02 MPa is depicted in Figure 29. NGH phase equilibrium temperature is determined to be 274.3 K. This temperature signifies the point at which NGH phase is in equilibrium.

**Figure 30:**
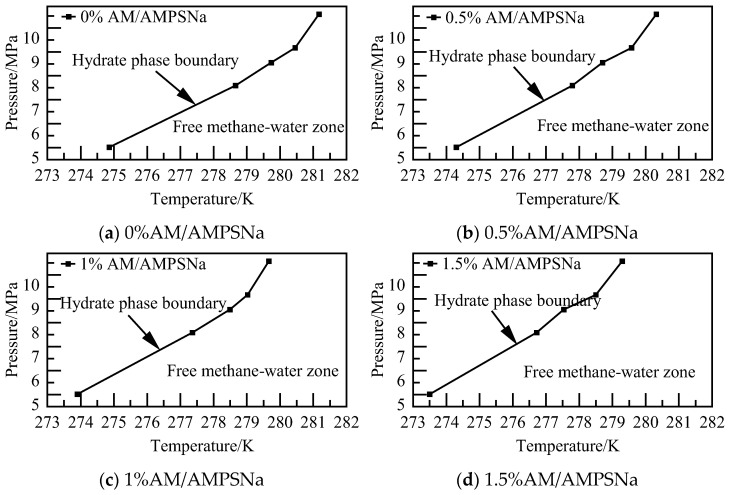
Diagram of NGH phase equilibrium.

The phase equilibrium calculations for the AM/AMPSNa system with various concentrations at different pressures are illustrated in Figure 30. As the solution concentration increases, the area where water molecules can retain the cage structure of NGH diminishes, while the region where water molecules exist as liquid water expands. In the case of the 1.5% AM/AMPSNa system, NGH phase equilibrium temperature is found to be lower compared to that of the pure water–NGH system, with a decrease ranging from 1.36 to 2.20 K.

#### 3.2.4. AA/AMPSNa/AM

**Figure 31:**
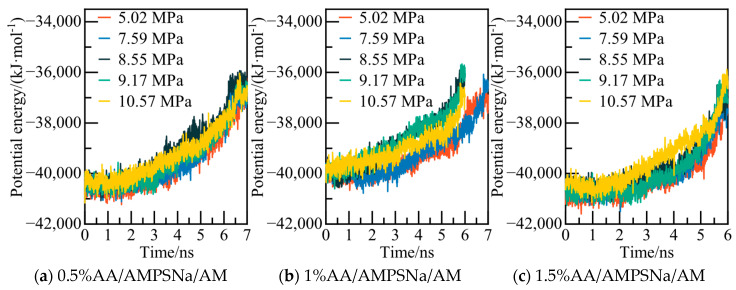
Potential energy changes at different pressures.

**Figure 32:**
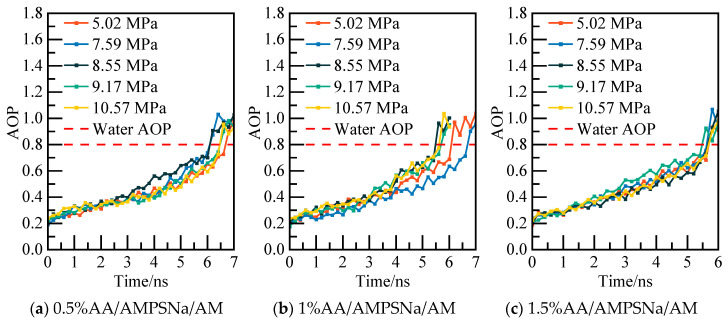
AOP changes of water molecules at different pressures.

The potential energy of the AA/AMPSNa/AM system and the AOP (average order parameter) value of water molecules are displayed in Figure 31 and Figure 32. For instance, considering the model of the 0.5% AA/AMPSNa/AM system at 5.02 MPa, the potential energy of the system stabilizes and reaches equilibrium at 6.8 nanoseconds. At this point, the corresponding AOP value exceeds 0.8, indicating that NGH has undergone complete decomposition.

**Figure 33:**
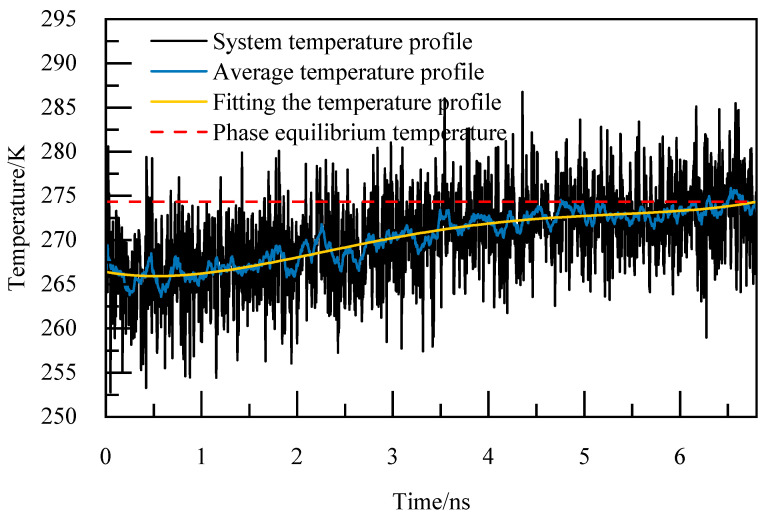
Temperature curves at 5.02 MPa.

In the case of the 0.5% AA/AMPSNa/AM system modeled at 5.02 MPa, the temperature profile was calculated and is depicted in Figure 33. NGH phase equilibrium temperature is determined to be 274.35 K, as indicated in the figure.

**Figure 34:**
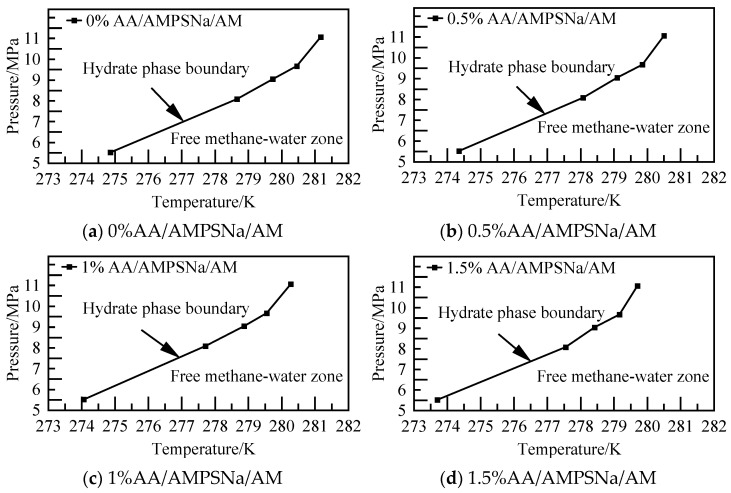
Diagram of NGH phase equilibrium.

The findings from the phase equilibrium calculations for the AA/AMPSNa/AM system at varying concentrations and pressures are illustrated in Figure 34. It is observed that with an increase in solution concentration, the area where NGH crystals can be stabilized diminishes, while the region where water molecules remain in a liquid state expands. Additionally, for the 1.5% AA/AMPSNa/AM system, NGH phase equilibrium temperature is lowered by 1.11–1.47 K in comparison to that of the pure water–NGH system.

For AM/AA, AA/AMPSNa, AM/AMPSNa, and AA/AMPSNa/AM, AM/AA had a stronger promotion effect on NGH decomposition than AA/AMPSNa, AM/AMPSNa, and AA/AMPSNa/AM under the same conditions.

When the cement slurry percolates, organic admixtures with varying structures induce a shift towards lower temperatures in NGH phase equilibrium, leading to the destabilization and decomposition of NGH, releasing methane gas. The variation in the type and quantity of functional groups present in the admixtures, along with their distribution on the carbon chain, influences the interaction of these admixtures on the NGH surface. Therefore, when structurally designing cementing additives, it is crucial to consider the impact of amide group activity and the synergistic effect of amide and carboxyl groups on NGH stability. This approach aims to mitigate the impact of the cementing process on NGH and ensure operational safety.

## 4. Conclusions

This paper established a NGH phase equilibrium calculation method under the influence of polymer additives in cement slurry filtrate using molecular simulation. Furthermore, the NGH phase equilibrium curve affected by polymer additives was characterized by this method. The following conclusions are drawn:

(1) LAMMPS software combined with the TIP4P model was utilized to construct an NGH phase equilibrium calculation method. Then, based on the single functional groups and combined functional groups in polymer additives, the potential energy, angular order parameter (AOP) of water molecules, and NGH phase equilibrium temperatures under different pressures were calculated. From the calculation results, the critical conditions for NGH stability were determined, and the phase equilibrium curve was characterized.
(2) The NGH phase equilibrium could be influenced by functional groups. For individual functional groups, the amide group exhibited the most significant impact on NGH phase equilibrium temperature. In the 1.5% C_12_H_25_ON system, the decrease in NGH phase equilibrium temperature ranged from 1.68 to 2.77 K. For combined functional groups, AM/AA exhibits the most significant adverse effects. In the 1.5% AM/AA system, the decrease in NGH phase equilibrium temperature ranged from 2.33 to 3.56 K.
(3) Through the calculation of NGH phase equilibrium and the characterization of phase equilibrium curves, guidance can be provided for the development of polymer additives in cement slurry for NGH formation cementing. Reducing the contents of amide groups and carboxyl groups in polymer additives is conducive to maintaining hydrate stability.

## Figures and Tables

**Table 1:** Model force field parameters of water molecules.

Molecular	Force Field Model	Atomic	*q*/e	*б*/nm	*ε*/(kcal·mol^−1^)
H_2_O	TIP4P	O	−1.04	0.31536	0.1550
		H	0.52	0	0

**Table 2:** Model force field parameters of methane molecules.

Molecular	Force Field Model	Atomic	*q*/e	*б*/nm	*ε*/(kcal·mol^−1^)
CH_4_	OPLS-AA	C	−0.3077	0.35	0.066
		H	0.0769	0.25	0.03

**Table 3:** Model force field parameters of solute molecules.

Force Field Model	Atomic	*q*/e	*б*/nm	*ε*/(kcal·mol^−1^)
OPLS-AA	-C in CH	−0.0114	0.35	0.066
	-H in CH	0.0114	0.25	0.03
	-C in C=O	0.6156	0.355	0.07
	-O in C=O	−0.3078	0.296	0.21
	-O in OH	−0.6270	0.312	0.17
	-N in NH	−0.3420	0.325	0.17
	H in -NH\-OH	0.3420	0	0
	-O in SO_3_M	−0.5130	0.296	0.21

**Table 4:** Equilibrium condition of water–NGH system.

Pressure/MPa	5.02	7.59	8.55	9.17	10.57
Temperature/K	274.86	278.66	279.73	280.45	281.17

## Data Availability

The original contributions presented in this study are included in the article. Further inquiries can be directed to the corresponding author.

## Nomenclature

NGH	natural gas hydrate
AOP	angular order parameter
C_12_H_24_O_2_	Dodecanoic acid
C_12_H_25_SO_3_Na	Sodium dodecyl sulfonate
C_12_H_25_ON	Dodecanamide
AA	acrylic acid
AM	acrylamide
AMPS	2-acrylamide-2-methylpropanesulfonic acid
AMPSNa	sodium 2-acrylamide-2-methylpropanesulfonate
